# Supplementary material for: MetaRibo-Seq measures translation in microbiomes
Source: Nat Commun. 2020 Jun 29;11:3268. doi: 10.1038/s41467-020-17081-z (PMC7324362; doi:10.1038/s41467-020-17081-z)
Supplement: Supplementary file 10 — Supplementary Data 7 [file 41467_2020_17081_MOESM10_ESM.zip › File2/Confidence_High_Taxonomy/98684_out.krona.html]

Javascript must be enabled to view this page.

members
magnitude
magnitudeUnassigned
count
unassigned
taxon
rank

98684\_out

2

2
superkingdom
2

32066
2
phylum

203490
2
class

2
order
203491

family
2
203492

848
genus
2

860

SRS1041114\_contig\_number\_contig-100\_12489.12490
species
1

species
1
851

76857

SRS049318\_contig\_number\_contig-100\_12657.291579
subspecies
1
